# Successful treatment of anti-glomerular basement membrane antibody disease combined with membranous nephropathy using rituximab: a case report

**DOI:** 10.3389/fimmu.2026.1886319

**Published:** 2026-10-28

**Authors:** Yaqian Cheng, Guojian Shao, LingJiu Shao, ZhouTao Xie, WenWen Hu, Binhui Pan, Yifan Zhang

**Affiliations:** Wenzhou Central Hospital, Affiliated to Wenzhou Medical University, Wenzhou, Zhejiang, China

**Keywords:** anti-glomerular basement membrane antibody disease, glucocorticoids, membranous nephropathy, plasma exchange, rituximab

## Abstract

Anti‑glomerular basement membrane (anti‑GBM) disease is a rare autoimmune disorder that classically manifests as rapidly progressive glomerulonephritis and pulmonary hemorrhage. The coexistence of anti-GBM disease and membranous nephropathy (MN) was first reported in 1976. Although such cases are rare, many reported patients develop progressive kidney injury and poor renal outcomes. We report a young man with sequential-onset dual glomerulopathy. He initially presented with MN alone and achieved partial remission following glucocorticoid and cyclosporine therapy. Three years later, he developed rapidly progressive glomerulonephritis, and kidney biopsy confirmed anti-GBM disease superimposed on pre-existing MN. This sequential pattern differs from the simultaneous onset of both lesions reported in many previous cases. To minimize gonadal toxicity, he received individualized therapy consisting of glucocorticoids, dose-adjusted cyclophosphamide, rituximab, and plasma exchange. He ultimately recovered normal kidney function and had a favorable renal outcome. This case expands the clinical spectrum of overlapping anti-GBM disease and MN and suggests that patients with MN in remission require long-term serial monitoring for early detection of superimposed autoimmune kidney injury. Individualized immunosuppressive strategies tailored to each patient’s clinical characteristics are essential to optimize kidney outcomes in such complex glomerular diseases.

## 1 Introduction

Anti-glomerular basement membrane (anti‑GBM) disease is a distinct small-vessel vasculitis that primarily affects the glomeruli and pulmonary capillaries, driven by autoantibodies directed against the α3 chain of type IV collagen. Renal histology typically reveals necrotizing and crescentic glomerulonephritis. The disease is rare, with an annual incidence of 0.60–1.79 per million population (1), and follows a rapidly progressive course associated with poor outcomes; one-year patient and renal survival rates are 69.4% and 37.7%, respectively (2). Membranous nephropathy (MN) is a common glomerular disease in adults, characterized clinically by nephrotic syndrome, whereas acute kidney injury is relatively uncommon. Renal pathology is characterized by subepithelial immune complex deposition, usually without crescent formation. Concurrent anti-GBM disease and MN are rarely reported, and affected patients often face a high risk of progression to end-stage renal disease despite standard immunosuppressive therapy. Here, we report a case of anti-GBM disease combined with MN in which the patient was successfully managed with rituximab.

## 2 Case report

A 24-year-old male patient of Han ethnicity (occupation: chef) presented to our hospital on August 31, 2022, with a chief complaint of bilateral lower extremity edema for three years and fatigue for one month. His medical history dates back approximately 7 years, when he developed facial and bilateral lower extremity edema following a period of staying up late, without accompanying oliguria, fever, or chest tightness. His past medical, personal, and family histories were unremarkable. He first presented to our hospital for further evaluation 7 years ago. On admission, laboratory tests revealed serum creatinine 73 μmol/L, urine occult blood (±), and urine protein (3+); serum albumin was 20.9 g/L; total cholesterol 12.24 mmol/L, triglycerides 2.29 mmol/L, low‑density lipoprotein cholesterol 8.54 mmol/L; 24‑hour urine protein 4.78 g; and anti‑phospholipase A2 receptor antibody was weakly positive (1:10). A full panel of antinuclear antibodies, anti-neutrophil cytoplasmic antibodies, immunofixation electrophoresis, and anti‑glomerular basement membrane antibodies were all normal. Six days later, the patient underwent a renal biopsy, which revealed stage II membranous nephropathy (**Figure 1**). He was treated with oral prednisone 50 mg once daily plus cyclosporine 100 mg twice daily. The patient reported regular medication adherence but did not attend regular follow‑up visits; his urine protein ranged from 1+ to 2+, and serum creatinine remained around 80 μmol/L.

**Figure 1:**
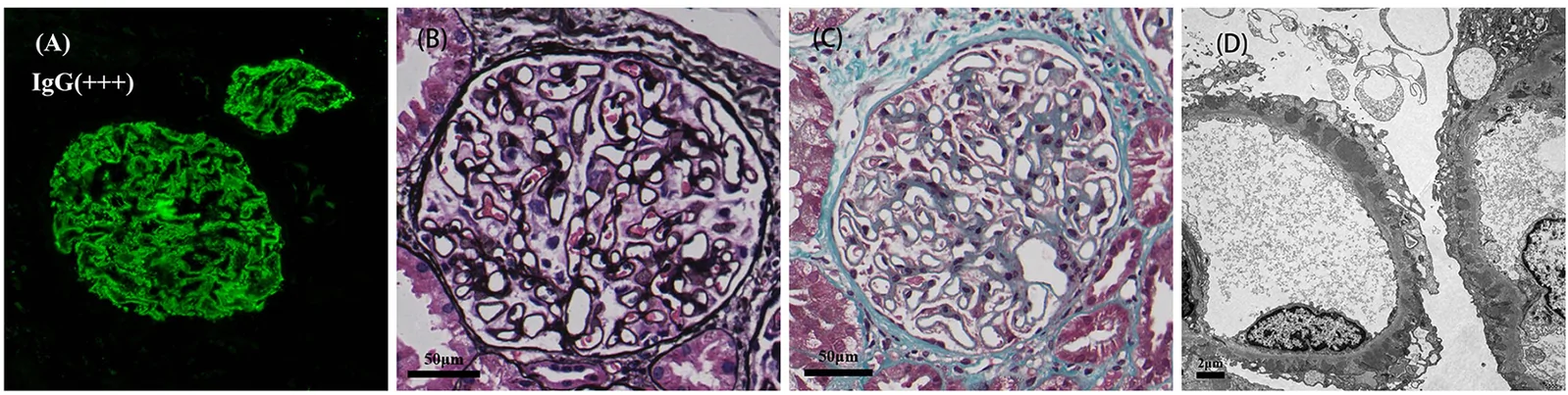
Pathological images from the first renal biopsy. **(A)** Immunofluorescence: diffuse, global, fine granular deposition of IgG along the glomerular capillary loops. **(B)** PASM staining: spike formation visible on the glomerular basement membrane. **(C)** Masson staining: subepithelial fuchsinophilic deposits. **(D)** Electron microscopy: numerous electron-dense deposits in the subepithelial and intramembranous regions.

One month before admission, following a period of sleep deprivation, the patient developed anorexia, accompanied by low-grade fever, cough, chest tightness, and generalized fatigue. He initially presented to the gastroenterology outpatient clinic of an external hospital. Contrast-enhanced abdominal computed tomography (CT) showed normal-appearing kidneys, a nonspecific finding near the left hepatic fissure, and a small amount of pelvic fluid. Repeat urinalysis showed urine protein (3+) and negative urine occult blood. The consulting nephrologist recommended an escalation in the dosage of glucocorticoids and cyclosporine. The patient subsequently presented to our hospital and was readmitted approximately 4 years ago. Physical examination on admission revealed the following: temperature 36.3 °C, pulse 99 beats/min, respiratory rate 20 breaths/min, blood pressure 122/71 mmHg, height 173 cm, weight 62 kg, and pallor; cardiopulmonary and abdominal examinations were unremarkable, and there was no lower extremity edema.

Upon admission, comprehensive auxiliary examinations were conducted. Routine blood tests showed a white blood cell count of 9.9×10^9^/L, hemoglobin 84 g/L, and platelet count 510×10^9^/L. Urinalysis revealed occult blood 1+, red blood cells 3201.0/μL, protein 2+, trace leukocyte esterase, and white blood cells 30.0/μL. Blood biochemistry demonstrated hypoalbuminemia (albumin 23.3 g/L), elevated urea (8.7 mmol/L), creatinine(134 μmol/L), low-density lipoprotein cholesterol (3.38 mmol/L), and C-reactive protein (31.8 mg/L).The anti-phospholipase A2 receptor antibody(PLA2R) titer was 25.28 RU/mL, and 24-hour urine protein excretion was 5.22 g.

A comprehensive workup for secondary causes and concurrent infections was negative, including tests for immunoglobulins, complement, serum free light chains, antinuclear antibody (ANA) panel, anti-neutrophil cytoplasmic antibodies (ANCA), hemolytic markers, Coombs test, tumor markers, and a standard transfusion-transmitted infection panel. Concurrently, anti-GBM antibody testing was performed, but the results were still pending. Furthermore, screenings for bacterial, fungal, and viral pathogens—including blood cultures, (1,3)-β-D-glucan, galactomannan, T-SPOT.TB, influenza A/B, cytomegalovirus, Epstein-Barr virus, and SARS-CoV-2— revealed no significant abnormalities. Chest CT showed a small nonspecific nodule in the right lower lobe, without clear clinical significance.

During hospitalization, the patient received symptomatic treatments, including albumin infusion, enoxaparin for anticoagulation, alprostadil to improve renal circulation, and erythropoietin to correct anemia. Subsequently, he developed febrile episodes accompanied by chills, with a peak temperature of 38.4 °C, but without cough or hemoptysis. Because infection was initially suspected, empirical intravenous piperacillin–tazobactam was administered; however, subsequent evaluation revealed no evidence of infection, and the fever was attributed to immune activity. By day 4 of admission, his serum creatinine showed a progressive increase to 188 μmol/L. Consequently, intravenous fluid support was administered, and renin-angiotensin-aldosterone system inhibitors along with cyclosporine were discontinued.

On day 9, repeat laboratory testing revealed a further sharp rise in serum creatinine to 372 μmol/L. On day 10, the result returned positive for anti‑GBM IgG, confirming a diagnosis of concurrent anti-GBM disease. Therapeutic plasma exchange (TPE) was initiated immediately using fresh frozen plasma as the replacement fluid, with a replacement volume of 45 mL/kg, a blood flow rate of 100–120 mL/min, and a replacement fluid flow rate of 1000 mL/h. Concurrently, the immunosuppressive regimen included intravenous methylprednisolone (80 mg once daily for 3 days), followed by oral methylprednisolone (48 mg once daily), and intravenous cyclophosphamide 0.8 g (with an additional 0.2 g given one week later).

During this period, a second renal biopsy was performed. Immunofluorescence examination of the biopsy specimen revealed 10 glomeruli with diffuse capillary loop staining for IgG (3+), IgG1 (±), IgG2 (+~++), IgG3 (+), IgG4 (4+), IgM (+), C3 (++~+++), C1q (±), and PLA2R (+), demonstrating global, granular deposition. Staining for IgA, fibrinogen, and THSD7A was negative and no immunoglobulin or complement deposition was observed in extraglomerular structures. Light microscopy identified 15 glomeruli, of which 10 contained cellular fibrous crescents of varying sizes, most with loop necrosis. In the remaining glomeruli, focal segmental mild proliferation of mesangial cells and matrix was observed. Among the glomeruli without crescents, two showed segmental loop necrosis. PASM staining demonstrated numerous spike‑like structures on the epithelial side of the glomerular capillary loop basement membrane. Tubular epithelial cells exhibited vacuolar and granular degeneration; protein and red blood cell casts, as well as occasional inflammatory cell casts, were observed. Some tubules showed luminal dilatation, loss of brush border, formation of bare basement membrane, and focal atrophy; occasional shedding of epithelial cells into the lumen was also seen. The renal interstitium appeared edematous, with diffuse infiltration of lymphocytes and mononuclear cells, scattered plasma cells and neutrophils, and moderate‑to‑severe interstitial fibrosis. Electron microscopy of one glomerulus revealed abundant electron\‑dense deposits in the subepithelial and intramembranous regions of the capillary loop basement membrane, with lucent areas around intramembranous electron deposits in segments (**Figure 2**). Based on the clinical data and renal biopsy findings, the final diagnoses were rapidly progressive glomerulonephritis, anti‑GBM disease, and stage III membranous nephropathy.

**Figure 2:**
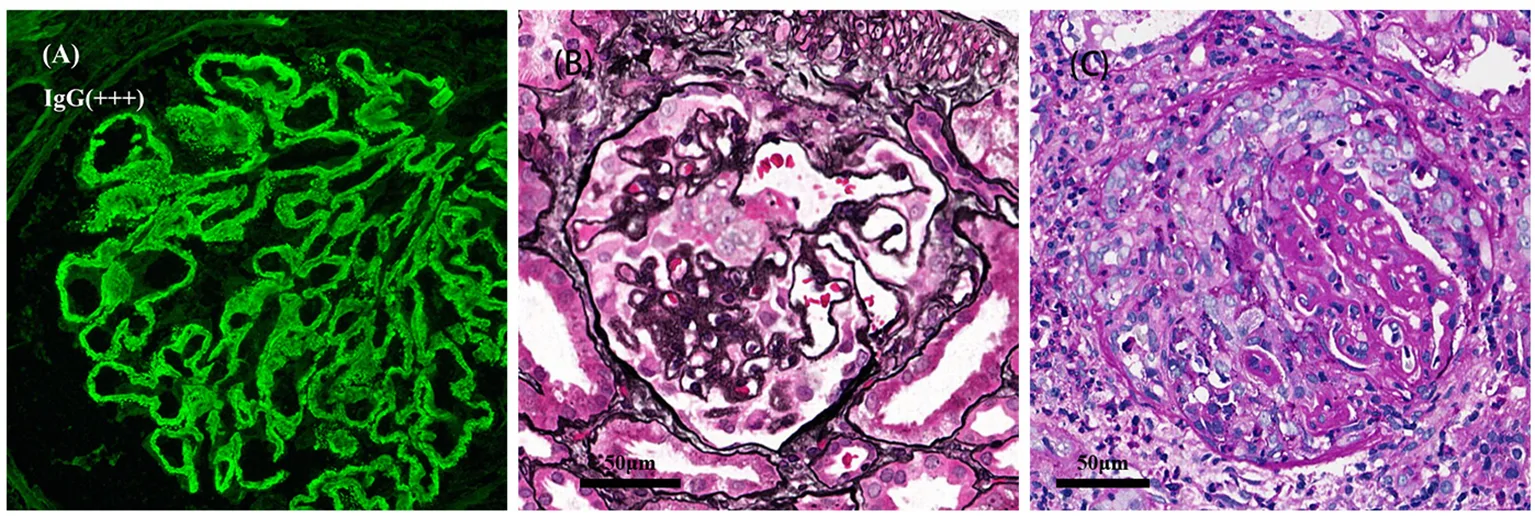
Pathological images from the second renal biopsy. **(A)** Immunofluorescence: diffuse, global, fine granular deposition of IgG along the glomerular capillary loops. **(B)** PASM staining: loop necrosis. **(C)** PAS staining: crescent formation.

On day 16, the patient’s clinical condition deteriorated, with serum creatinine rising to 773 μmol/L, and 24-hour urine protein excretion increasing to 16.87 g. Consequently, an intensified immunosuppressive regimen was initiated: oral methylprednisolone (48 mg/day) was discontinued, and intravenous methylprednisolone pulse therapy was started at 500 mg/day for 3 days, followed by intravenous methylprednisolone 80 mg/day for 3 days, before transitioning back to oral methylprednisolone (40 mg once daily). Cyclophosphamide was replaced with rituximab given the patient’s fertility concerns. Rituximab was initiated intravenously at 600 mg on day 20, followed by three additional 300-mg doses on days 30, 40, and 47. A total of 14 sessions of therapeutic plasma exchange was completed. Supportive treatments included intravenous immunoglobulin, gastric protection, calcium supplementation. Prophylaxis against opportunistic infections was provided with voriconazole (for fungal infections) and trimethoprim-sulfamethoxazole (for *Pneumocystis jirovecii* pneumonia). Serial laboratory assessments demonstrated a favorable response: serum creatinine gradually decline to 100 μmol/L, and the patient achieved serological remission, with both anti-GBM and anti-phospholipase A2 receptor antibodies undetectable. Glucocorticoids were gradually tapered, and azilsartan was added to reduce proteinuria. After the patient’s condition stabilized, he was discharged, at which time the oral methylprednisolone dose had been reduced to 28 mg once daily.

Following discharge, the patient was regularly followed up at the outpatient clinic, where glucocorticoids were gradually tapered according to the prescribed regimen and discontinued six months later. Throughout treatment, lymphocyte counts and immunoglobulin levels were monitored regularly. Under close follow-up, IgG levels remained persistently above 3 g/L. Although the CD4+ T-cell count transiently fell below 200 cells/μL, the patient continued to receive prophylactic anti-infective therapy, and no infection-related adverse events occurred. At the last follow-up on May 28, 2025, he was asymptomatic and receiving azilsartan, dapagliflozin, and finerenone. Serum creatinine was 74 μmol/L, 24-hour urine protein excretion was 0.55 g, and both anti-phospholipase A2 receptor and anti-glomerular basement membrane antibodies were negative. No adverse events occurred throughout the entire treatment course. The treatment outcome was deemed satisfactory. **Figure 3** shows the trends in the patient’s serum creatinine level and 24-hour urine protein excretion.

**Figure 3:**
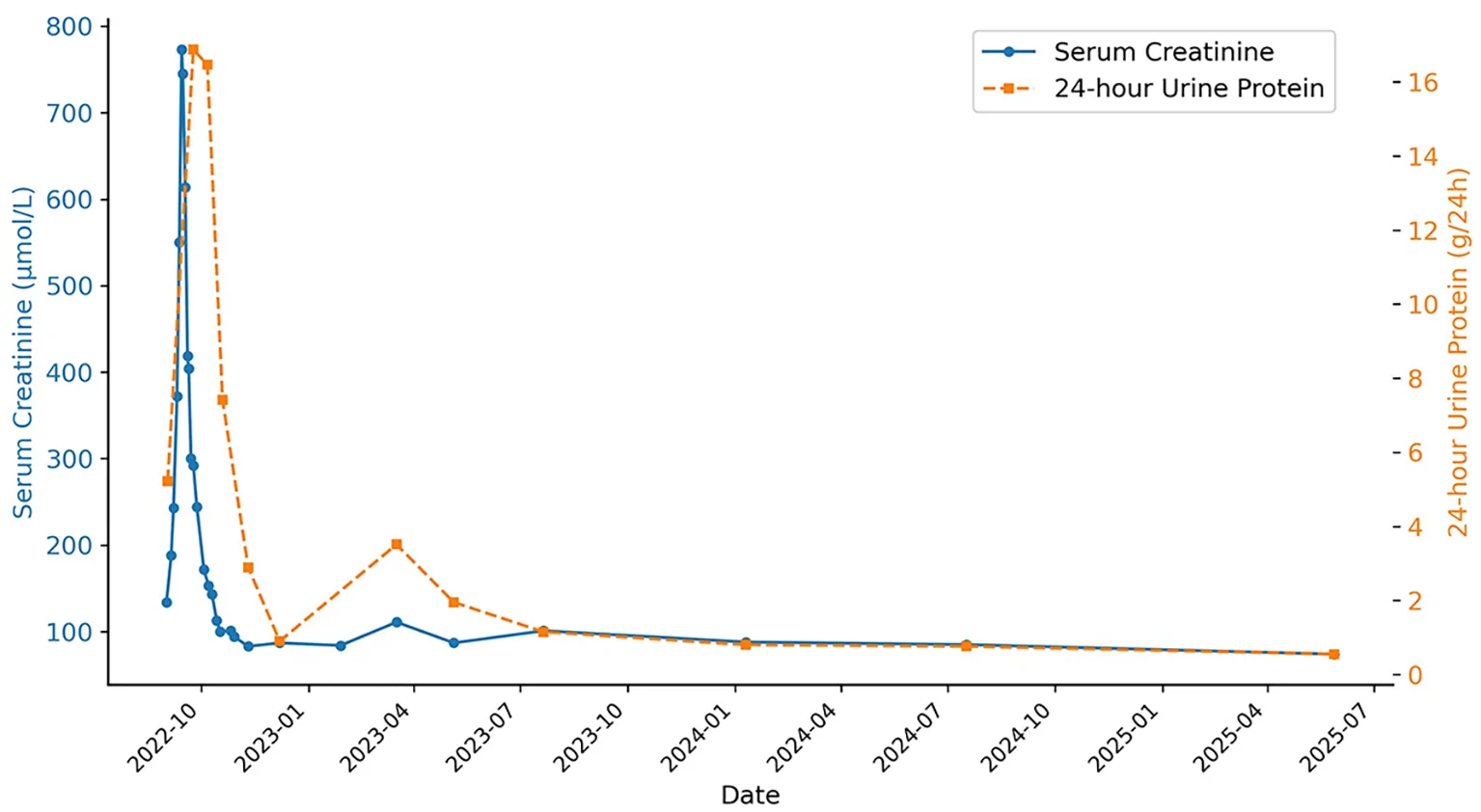
Trends in the patient’s serum creatinine and 24-hour urine protein levels.

## 3 Discussion

Anti-GBM disease typically presents as a distinct clinical entity but can occasionally overlap with other glomerulopathies. While concurrent anti-neutrophil cytoplasmic antibody (ANCA)-associated vasculitis is the most common; the coexistence of anti-GBM disease with MN is exceptionally rare. Herein, we report a case of a 24-year-old male who initially presented with nephrotic syndrome, tested positive for anti-phospholipase A2 receptor antibody, and was histologically diagnosed with MN. Despite an initial treatment with glucocorticoids and cyclosporine, the patient achieved clinical partial remission of MN. Three years later, he developed rapidly progressive glomerulonephritis (RPGN) and was found to have dual seropositivity for both anti-GBM and anti-phospholipase A2 antibodies. A subsequent renal biopsy confirmed the diagnosis of anti-GBM disease combined with MN. Following prompt and intensive immunosuppressive therapy-including glucocorticoids, cyclophosphamide, rituximab, and therapeutic plasma exchange-the patient achieved complete recovery of renal function and partial remission of MN. This case underscores that early diagnosis and timely, aggressive intervention are pivotal for improving prognosis.

The precise pathogenesis underlying the overlap of anti-GBM disease and MN remains elusive. One prevailing hypothesis posits that immune complex deposition characteristic of MN induces structural damage to the glomerular basement membrane (GBM). This injury may expose previously sequestered autoantigens within the GBM to the systemic circulation, thereby triggering the production of anti-GBM antibodies. Kurki et al. reported a patient who developed anti-GBM disease one year after being diagnosed with MN. Retrospective analysis of blood samples collected during follow-up revealed that serum anti-GBM antibody levels had begun to rise two months before the decline in renal function (3). This finding highlights the critical importance of close monitoring of anti-GBM antibodies during the long-term follow-up of patients with MN, as it may facilitate the early detection and management of this rare but severe complication.

The current therapeutic strategy for concurrent anti-GBM disease and MN generally adheres to the classic anti-GBM protocol, which includes glucocorticoid pulse therapy, immunosuppressants, and intensive plasma exchange. Recently, rituximab has been increasingly used as an emerging therapy for anti-GBM disease. In one study, eight patients with anti‑GBM disease received rituximab added to the classic regimen; after a mean follow-up of 25.6 months, pulmonary hemorrhage was completely controlled in seven patients, and overall survival was favorable; however, improvement in renal function was not significant (4). Other studies have shown that using rituximab as a first-line immunosuppressant, combined with glucocorticoid pulse therapy and plasma exchange, can achieve negativity for anti-GBM antibodies, enable most patients to discontinue hemodialysis, and is associated with no adverse reactions (5, 6). In addition, multiple studies have demonstrated that rituximab can accelerate renal function recovery and reduce anti-GBM antibody levels in patients with anti-GBM disease (7–10). In the present case, treatment was initiated immediately after diagnosis. On the basis of high-dose glucocorticoid pulse therapy, therapeutic plasma exchange, and two doses of intravenous cyclophosphamide, cyclophosphamide was switched to rituximab considering the patient’s young age and fertility requirements, as multiple lines of evidence support the beneficial effects of rituximab in anti-GBM disease. Subsequently, the patient’s serum creatinine gradually decreased, reaching 100 μmol/L one month later, which was close to the normal range.

Regarding the prognosis of concurrent anti-GBM disease and MN, no consensus has been reached in the current literature. The largest case series to date included 12 patients with anti-GBM disease and MN. Among them, eight received glucocorticoids, cyclophosphamide, and plasma exchange; two received methylprednisolone and plasma exchange; and two received rituximab. Ultimately, ten patients required renal replacement therapy, and only two recovered renal function (11), suggesting that patients with this dual diagnosis generally face a dismal renal outcome. In contrast, another observational study compared 13 patients with anti-GBM disease alone and seven with anti-GBM disease combined with MN, and found that the combined group had fewer acute kidney injury events than the anti-GBM-alone group, a lower incidence of crescents (14.3%), and better one-year renal survival (12). The higher renal survival rate in that study is speculated to be related to the lower incidence of crescents in the cohort, as crescents in other reports often portend an unfavorable outcome, with patients requiring long-term dialysis despite treatment with glucocorticoids, cyclophosphamide, and plasma exchange (13). In our case, the patient presented with rapid progression of renal dysfunction and a high number of crescents, suggesting a guarded prognosis. However, after early aggressive treatment, he achieved complete recovery of renal function and a marked decrease in proteinuria. After more than three years of follow-up, there has been no evidence of disease relapse or adverse events.

Several factors likely contributed to the favorable outcome in this case: timely diagnosis and treatment before irreversible renal injury, young age and absence of comorbidities or extrarenal manifestations (e.g., pulmonary hemorrhage), prompt initiation of a multimodal regimen (glucocorticoids, plasma exchange, cyclophosphamide, and rituximab), and the absence of adverse events throughout therapy. Additionally, the delayed development of anti‑GBM disease allowed early intervention after the acute rise in serum creatinine. These factors collectively explain why this patient achieved complete renal recovery, in contrast to the generally poor prognosis reported in the literature.

This report has several limitations that warrant consideration. First, this is a single case study, which limits the generalizability of our findings. Second, although the patient achieved complete renal function recovery and remained stable during more than three years of follow‑up, the long‑term risk of disease relapse or progression to chronic kidney disease remains unknown. Third, the precise temporal relationship between the onset of membranous nephropathy and the subsequent development of anti‑GBM disease could not be definitively established, as serial anti‑GBM antibody monitoring was not performed before the acute deterioration. Finally, the optimal dosing and duration of rituximab therapy in this dual‑disease setting remain unclear, and further prospective studies are required to validate our observations.

In summary, the coexistence of anti-GBM disease and MN is uncommon in clinical practice. For patients with MN, close monitoring of anti-GBM antibodies and repeat renal biopsy when indicated are essential to facilitate early diagnosis and treatment. Rituximab may be considered as an alternative option for those who respond poorly to conventional therapy. This case suggests that even when renal biopsy shows extensive crescent formation, early aggressive treatment may still lead to favorable renal outcomes.

## Data Availability

The original contributions presented in the study are included in the article/**Supplementary Material**. Further inquiries can be directed to the corresponding author.
